# Supramolecular Lubricating Hydrogel Microspheres Reshape Damaged Matrix Regeneration

**DOI:** 10.1002/advs.202504319

**Published:** 2025-07-16

**Authors:** Hui Yuan, Pengcheng Xiao, Wei Huang, Wenguo Cui

**Affiliations:** ^1^ Department of Orthopedics Shanghai Key Laboratory for Prevention and Treatment of Bone and Joint Diseases Shanghai Institute of Traumatology and Orthopedics Ruijin Hospital Shanghai Jiao Tong University School of Medicine 197 Ruijin 2nd Road Shanghai 200025 P. R. China; ^2^ Department of Orthopedics The First Affiliated Hospital of Chongqing Medical University Orthopedic Research Laboratory Chongqing Medical University Chongqing 400016 P. R. China

**Keywords:** damaged matrix regeneration, hydrogel microspheres, lubrication factor regeneration, lubrication supplementation, supramolecular lubrication

## Abstract

Supramolecular lubrication refers to lubricant supplementation utilizing intermolecular noncovalent interactions to remodel lubrication structure at biological interfaces. The previous studies found that lubrication supplementation is closely related to damaged matrix regeneration, but the lubrication structure is prone to disintegration and failure. Here, combining with microfluidic and photopolymerization strategies, a supramolecular lubricating hydrogel microsphere is constructed, which is medicated by dipalmitoylphosphatidylcholine (DPPC) liposomes as the core, a natural component in body fluids, and complexed with cartilage matrix‐binding peptide functionalized methacryloylated hyaluronan acid (WYR‐HAMA) as the photoinitiated site. The highly intermolecular ion‐dipole interaction between DPPC and WYR‐HAMA can form a dynamic supramolecular lubricating layer on the damaged matrix interfaces, where inflammatory factor inhibitors are coupled with DPPC liposomes via hydrophobic interaction to resist inflammatory transmission. Additionally, microspheres can autonomously regulate lubrication structure: in the early and middle stages, they can actively provide microscale lubrication (µ ≈ 0.04); in the late stage, the wear debris induced by endogenous enzymatic decomposition transmitted to provide nanoscale lubrication for the damaged matrix (µ ≈ 0.06). In vivo experiments demonstrate microspheres promoted efficient extracellular matrix synthesis and the autonomous release of lubrication factors at the cellular level. The developed bioplatform allowing cellular lubrication metabolism would provide a promising strategy for regenerating friction‐induced matrix damage.

## Graphical abstract

The supramolecular lubricating hydrogel microspheres can provide stable mechanophysical protection at damaged matrix through interfacial hydration lubrication: in the early and middle stages, microspheres can actively provide microscale supramolecular lubrication (µ ≈ 0.04) for damaged matrix; in the late stage, the wear debris induced by shear and endogenous enzymatic decomposition transmitted to provide nanoscale supramolecular lubrication for damaged matrix (µ ≈ 0.06).

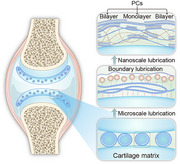

## 1 Introduction

The freedom of human activity benefits from supramolecular lubrication behavior, which enables rapid response to various mechanical stresses at biological interfaces.^[^ ^1^ ^]^ Supramolecular lubrication refers to a lubricant supplementation technology that utilizes intermolecular noncovalent interactions to regenerate damaged matrix, thereby establishing a stable supramolecular lubrication structure at biological interfaces.^[^ ^2^ ^]^ Research has demonstrated that the surfaces of lubrication biomaterials could mimic the interfacial characteristic of natural tissues, serving as a frictional supplementation to resist mechanical and physical damage.^[^ ^2c^ ^]^ Clinical studies also indicated supramolecular lubrication therapy could reduce friction between tissues and accelerate damaged matrix repair.^[^ ^3^ ^]^ Therefore, it is necessary to perform supramolecular lubrication supplementation on damaged matrix for enhancing their lubrication metabolism at the cellular level, so as to achieve damaged matrix regeneration in vivo.

Various supramolecular lubrication strategies based on biomaterials have been applied to regenerate damaged matrix. Zhang et al. synthesized a shear responsive lubrication supramolecular hydrogel through a thixotropic supramolecular and polymer double network.^[^ ^4^ ^]^ Similarly, Wang et al. created a dynamically repaired supramolecular lubricating surface through polymer monolayers self‐assembly, which relied on host‐guest interactions. Under frictional conditions, the host‐guest complexes preferentially dissociated, allowing the surfaces to recover from mechanical wear and restore lubricity by reforming noncovalent bonding interactions.^[^ ^5^ ^]^ Therefore, supramolecular hydrogels or surfaces are considered optimal lubrication biomaterials for damaged matrix. However, these supramolecular hydrogels or surfaces are susceptible to significant losses, including volatilization and degradation due to molecular diffusion, mechanical external forces and fluid shear, which compromise their long‐term lubrication efficacy. Currently, strategies such as crosslinking, chemical grafting, biomimetic methods, or increasing molecular weight were employed to improve the anti‐friction performance of lubricants.^[^ ^6^ ^]^ Nevertheless, these methods do not fundamentally address the loss of supramolecular lubrication structures and the generation of wear debris. The unique supramolecular lubrication property at biological interfaces is a complex and dynamic process, suggesting that lubricants should engage with damaged matrix in a more natural manner.^[^ ^4^, ^7^ ^]^ Therefore, effectively simulating the dynamic supramolecular lubrication behavior at damaged matrix remains a challenge in the design and synthesis of artificial lubricants. Consequently, the development of a hydrogel friction site with self‐regulating supramolecular lubrication structure is essential for promoting the regeneration of damaged matrix.

Osteoarthritis (OA) respents the most prevalent degenerative disease resulting from a failure in lubrication, which is characterized by the progressive degradation of the supramolecular lubrication structure.^[^ ^8^ ^]^ This degradation primarily arises from alterations in biomacromolecules, specifically the detachment of lubricating factors within the compromised cartilage matrix, which is unable to autonomously reconstruct the supramolecular lubrication structure for cartilage protection.^[^ ^9^ ^]^ Consequently, there is an urgent demand to develop a joint repair formulation capable of self‐remodeling the supramolecular lubrication structure at the interface of the damaged matrix. We used microfluidic technology to construct hyaluronic acid (HA) hydrogel and coated the surface with hydrogenated soybean phosphatidylcholine liposomes to improve its lubricating ability. However, HA in hydrogels was easily degradation by endogenous enzymes in joints, leading to partial or complete disintegration of lubrication structure of HA and liposomes, and ultimately completed lubrication failure.^[^ ^10^ ^]^ As a result, these hydrogel microspheres were still inadequate in reshaping the supramolecular lubrication structure. Cartilage lubrication is characterized by a highly dynamic interfacial supramolecular lubrication process. Therefore, a critical challenge lies in the re‐enrichment of the lost supramolecular lubricating fluid into the damaged matrix network, which may facilitate the restoration of lubrication at the damaged matrix interface.

**Figure 1:**
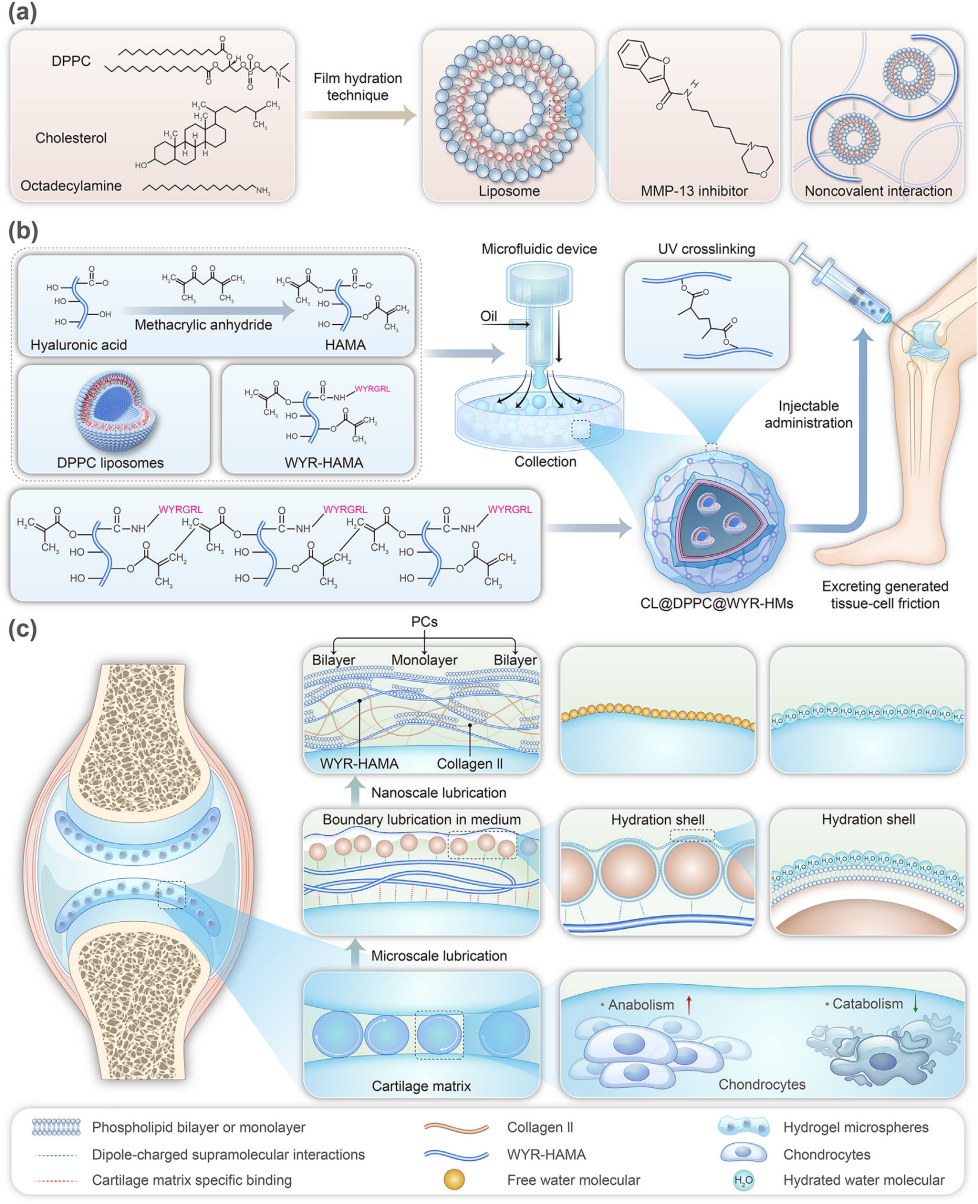
The fabrication progress of CL@DPPC Lipo@WYR‐HMs. a) The fabrication of the CL@DPPC Lipo. b) The fabrication and crosslinking of CL@DPPC Lipo@WYR‐HMs. (c) The combining rolling and hydration lubrication mechanism of the CL@DPPC Lipo@WYR‐HMs.

Inspired by the dynamic supramolecular lubrication interaction at natural tissue interfaces, we developed a supramolecular lubrication hydrogel microspheres supplementation site (**Figure** **1**). This site was composed of dipalmitoylphosphatidylcholine (DPPC) liposomes as the core, a natural component in human body fluids, and was complexed with cartilage matrix‐binding peptide (WYR) functionalized methacryloylated hyaluronan acid (WYR‐HAMA) as the photoinitiated site. The high intermolecular affinity between DPPC liposomes and WYR‐HAMA facilitates the formation of a supramolecular lubrication layer through ion‐dipole interaction on the surface of damaged matrix. Furthermore, inflammatory factor inhibitors (CL‐82198, CL) were coupled with DPPC liposomes via hydrophobic interactions to impede inflammatory transmission during the regeneration of the damaged cartilage matrix. Additionally, the microspheres possess the ability to autonomously regulate the lubrication structure: during the early and middle stages, the microspheres actively provide microscale supramolecular lubrication for the damaged matrix; in the later stages, wear debris induced by endogenous enzymatic decomposition contributes to the provision of nanoscale supramolecular lubrication for the damaged matrix. In vivo experiments demonstrated that the supramolecular lubrication hydrogel microspheres stimulated the efficient synthesis of extracellular matrix and the autonomous secretion of supramolecular lubrication factors at the cellular level, thereby promoting the in‐situ regeneration of the damaged matrix.

## 2 Results and Discussion

### 2.1 Preparation and Characterization of Supramolecular Lubricating Hydrogel Microspheres

HA was chosen as the structural component for HAMA microspheres (HMs) due to its role as a constituent of the extracellular matrix,^[^ ^11^ ^]^ which imparts favorable biocompatibility and lubrication characteristics.^[^ ^12^ ^]^ Furthermore, HA molecules can interact noncovalently with liposomes, resulting in the formation of a liposome reservoir that enhances the exposure of the highly hydrated phosphatidylcholine moiety within the liposomes, thereby establishing a stable hydrated lubricating layer.^[^ ^1b^ ^,^ ^10^ ^,^ ^13^ ^]^ The supramolecular lubricating hydrogel microspheres were synthesized by photocrosslinking WYR‐functionalized methacrylic anhydride (MA)‐modified HA (WYR‐HAMA). The results of 1H NMR spectroscopy, as illustrated in Figure S1 (Supporting Information), reveal the emergence of new signals at 5.7 and 6.1 ppm, indicative of successful MA modification, alongside signals in the range of δ = 7.04–6.90, confirming successful grafting of WYR. The degree of methacrylatization and WYR substitution, determined by comparing the peak at 1.9 ppm, were found to be 59% and 22%, respectively. Additionally, UV‐visible spectroscopy was employed to further validate the successful synthesis of cartilage matrix‐binding peptide functionalized hyaluronan acid (WYR‐HA), with a single absorption peak observed at 278 nm in WYR‐HA, absent in HA (Figure S2, Supporting Information), further supporting the successful grafting of WYR onto the HA backbone.

**Figure 2:**
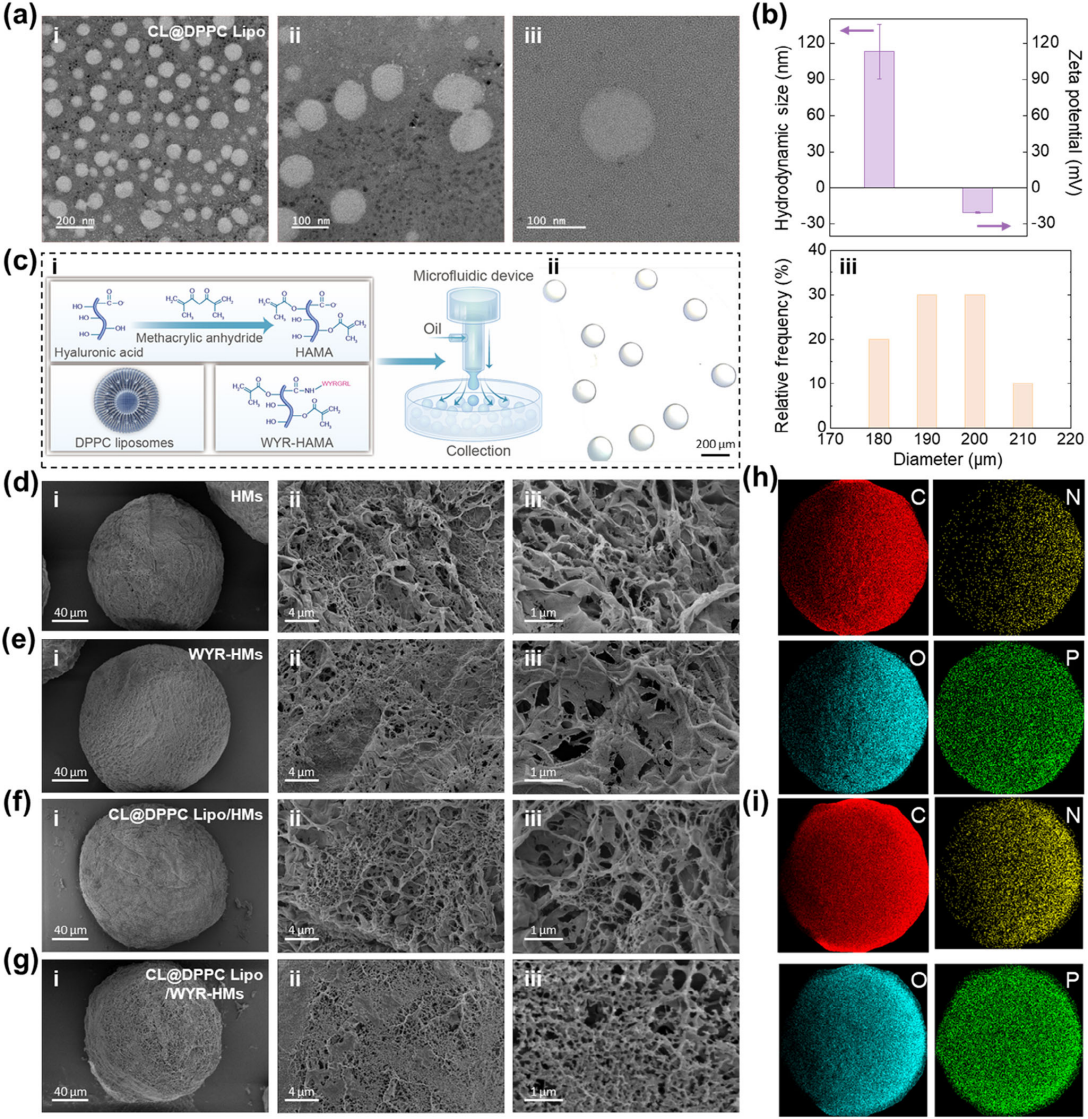
Characterization of CL@ DPPC Lipo and CL@ DPPC Lipo@WYR‐HMs. a) TEM of CL@DPPC Lipo. b) Hydrodynamic size and zeta potential of CL@DPPC Lipo. c) (i) The fabrication, (ii), and (iii) size distribution of microfluidic CL@ DPPC Lipo@WYR‐HMs. d) SEM image of HMs at the scale of (i) 40 µm, (ii) 4 µm and (iii) 1 µm. e) SEM image of WYR‐HM at the scale of (i) 40 µm, (ii) 4 µm, and (iii) 1 µm. f) SEM image of CL@ DPPC Lipo@HMs at the scale of (i) 40 µm, (ii) 4 µm, and (iii) 1 µm. g) SEM image of CL@ DPPC Lipo@WYR‐HMs at the scale of (i) 40 µm, (ii) 4 µm, and (iii) 1 µm. h) EDS analysis of CL@ DPPC Lipo@HMs. (i) EDS analysis of CL@ DPPC Lipo@WYR‐HMs.

DPPC lipids, which are naturally occurring components of human body fluids,^[^ ^14^ ^]^ were utilized to prepare liposomes due to their robust properties that facilitate effective boundary lubrication under physiological high pressure and their ability to assemble with HMs to create supramolecular lubrication complexes.^[1a]^ Transmission electron microscopy (TEM) images of CL@DPPC Lipo, fabricated via the thin film hydration method, reveal a spherical vesicle (**Figure** **2** **a)**. As depicted in Figure 2b, CL@DPPC Lipo exhibited a negative zeta potential of ‐20.58 ± 0.53 mV, which enhances repulsive interactions between the liposomes and negatively charged cells in cartilage, thereby mitigating the risk of liposome phagocytosis during supramolecular assembly. The average size of CL@DPPC Lipo was measured at 113.16 ± 22.46 nm, with a polydispersity index (PDI) of 0.21, suggesting favorable dispersibility and stability. The small size of the CL@DPPC Lipo also facilitates an increased number of assembly sites with HMs.

In this investigation, supramolecular hydrogel microspheres were fabricated through the photopolymerization of pre‐gel droplets generated by microfluidic device under the 30 uL min^−1^ of water phase flow rate, 1500 uL min^−1^ of oil phase flow rate.^[^ ^15^ ^]^ Microscopic observations and scanning electron microscopy (SEM) analyses demonstrated that the hydrogel microspheres were well‐dispersed, measuring 190 ± 25 µm (Figure 2c), and exhibited a distinct micro/nano composite structure (Figure 2d–g). Energy dispersive spectroscopy (EDS) elemental analysis (Figure 2h,i) and X‐ray photoelectron spectroscopy (Figure S3, Supporting Information) revealed the presence of a phosphorus elemental (or peak) in CL@DPPC Lipo@HMs and CL@DPPC Lipo@WYR‐HMs, which was absent in HMs, thereby confirming the successful complexation of liposomes with WYR‐HMs.

### 2.2 Lubrication Evaluation of Supramolecular Lubricating Hydrogel Microspheres

**Figure 3:**
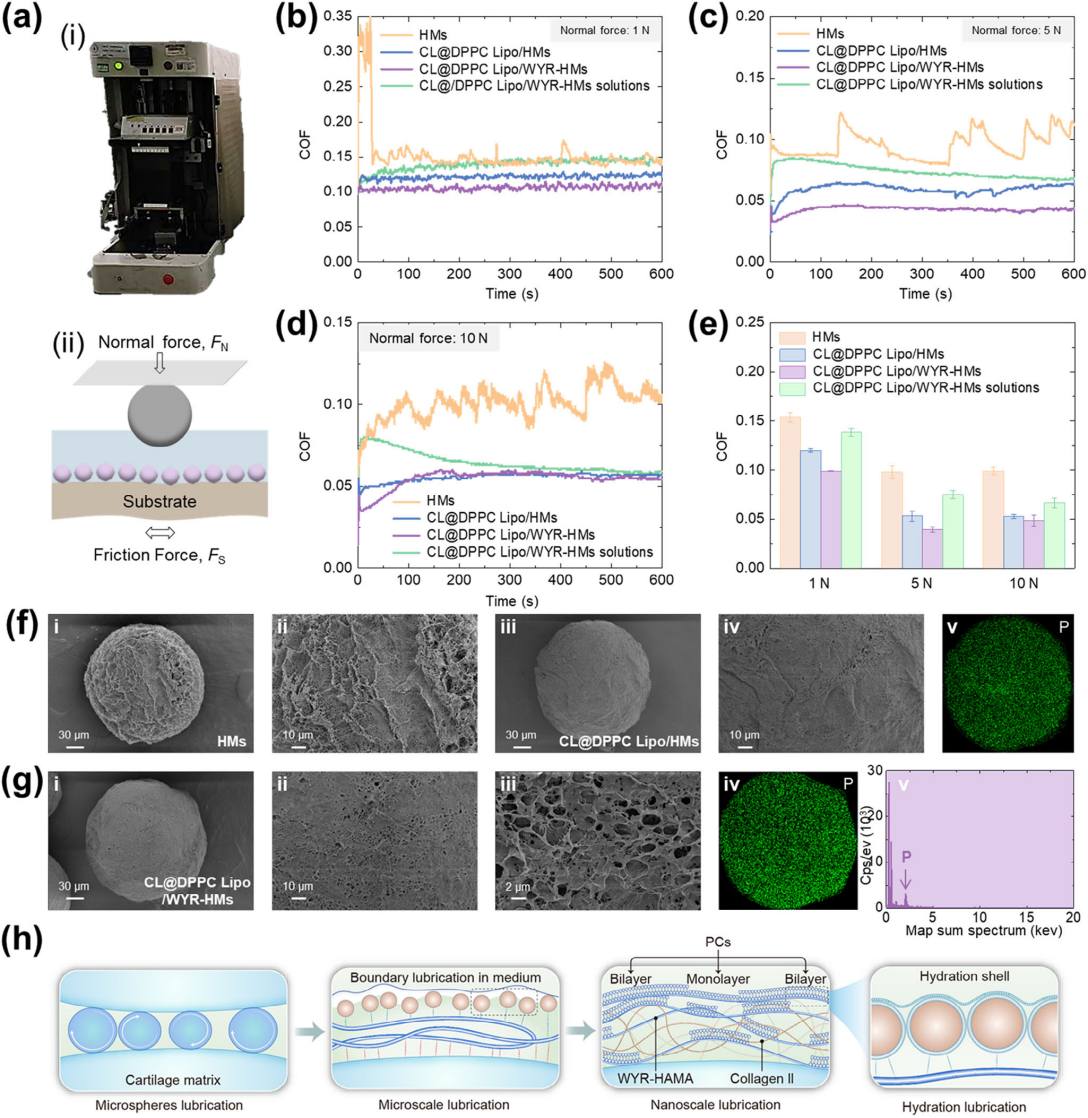
Lubrication performance of CL@DPPC Lipo@WYR‐HMs. a) (i) Photograph and (ii) schematic illustration of the friction measurement. b) The COF curve with time at 1 N of normal force. c) The COF curve with time at 5 N of normal force. d) The COF curve with time at 10 N of normal force. e) The average COF histogram at 1, 5, and 10 N. f) SEM images of prepared HMs (i and ii) and CL@DPPC Lipo@HMs (iii and iv; v was the P elements distribution after shear test). g) SEM images of prepared CL@DPPC Lipo@WYR‐HMs (i, ii, and iii; iv and v was the P elements distribution after shear test). h) The lubrication mechanism of CL@DPPC Lipo@WYR‐HMs.

To evaluate the lubrication properties of supramolecular hydrogel microspheres, linear friction tests were performed on various formulations, including HMs, CL@DPPC Lipo/HMs, CL@DPPC Lipo/WYR‐HMs, and CL@DPPC Lipo/WYR‐HMs solutions (mimicking the wear debris induced by shear and endogenous enzymatic decomposition) under different loading at a shear rate of 1 Hz (**Figure** **3**a). At a load of 1 N (corresponding to a maximum contact pressure of 25.68 MPa according to the Hertzian equation, approximating physiological joint pressure),^[6b,13a,16]^ the average COF were found to be similar across the different formulations: CL@DPPC Lipo/WYR‐HMs (0.10), CL@DPPC Lipo/HMs (0.12), HMs (0.15), and degraded CL@DPPC Lipo/WYR‐HMs molecules (0.14) (Figure 3b). When the load was increased to 5 N, simulating greater mechanical stress on diseased tissue, CL@DPPC Lipo/WYR‐HMs microspheres exhibited a lower COF (0.039) compared to CL@DPPC Lipo/HMs (0.053), HMs (0.098), and degraded CL@DPPC Lipo/WYR‐HMs molecules (0.075). At 10 N loading (Figure 3c), CL@DPPC Lipo/WYR‐HMs maintained a low COF (0.048), outperforming CL@DPPC Lipo/HMs (0.053) and HMs (0.099), while approaching the performance of degraded CL@DPPC Lipo/WYR‐HMs molecules (0.067) (Figure 3d). Figure 3e demonstrates an inverse correlation between COF and loading across all microspheres, with COF remaining below 0.15 even under higher loads. Importantly, these COF values are consistent with physiological ranges observed in biological systems: joint system <0.36.^[^ ^17^ ^]^ These findings affirm the lubrication properties of CL@DPPC Lipo/WYR‐HMs, particularly under elevated mechanical loads, indicating their potential effectiveness in minimizing friction.

Further analysis of COF‐time plots revealed distinct lubrication behavior among the microspheres. The COF values of HMs irregularly increased over shear time, which was not for CL@DPPC Lipo/HMs and CL@DPPC Lipo/WYR‐HMs. This indicates the lubrication film between the opposing surfaces at HMs could not be maintained and deactivated due to water loss, leading to an increase in COF. In contrast, the supramolecular coupling between DPPC liposomes and HMs formed a tighter hydration layer to resist shear force in CL@DPPC Lipo/HMs and CL@DPPC Lipo/WYR‐HMs.

To further validate the capacity of the hydrogel microspheres to withstand mechanical abrasion, surface morphology of HMs, CL@DPPC Lipo/HMs, and CL@DPPC Lipo/WYR‐HMs post‐friction was analyzed using SEM. As depicted in Figure 3f,g, liposomes were not detected on the outer surface of the DPPC Lipo@HMs in the random SEM field, while a phosphorus elemental (or peak) remained evident in CL@DPPC Lipo/WYR‐HMs. This observation indicates that the majority of the liposomes were encapsulated within WYR‐HMs, thereby endowing microspheres with a robust lubrication layer that effectively mitigates mechanical friction (Figure 3h). Our degradation studies (Figure S4, Supporting Information) also revealed a distinct biphasic degradation profile characterized by an initial gradual degradation phase over the first 28 days, followed by a subsequent rapid degradation phase that culminated in complete dissolution by day 48. This observed degradation pattern suggests that the microspheres undergo a structural change, transitioning from a microscale lubrication to a nanoscale phospholipid release lubrication, potentially mediated by hyaluronidase.

### 2.3 Supramolecular Lubricating Hydrogel Microspheres to Target Damaged Matrix

**Figure 4:**
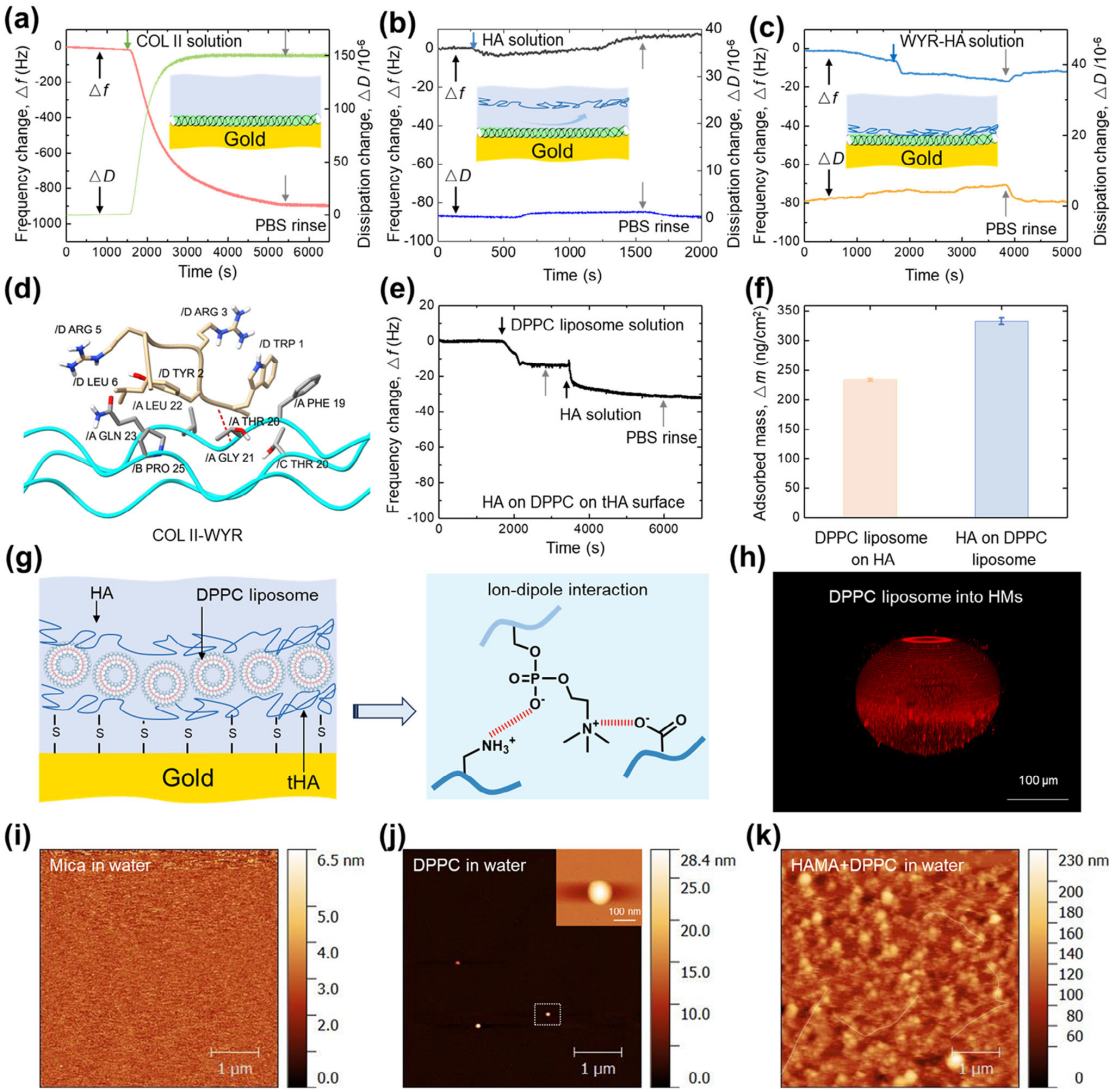
Supramolecular lubricating hydrogel microspheres to target damaged matrix and assemble behavior. a) The △*f* and△*D* plots for the adsorption of COL II on Au surface. b) The △*f* and△*D* plots for the adsorption of free HA on COL II surface. c) The △*f* and△*D* plots for the adsorption of WYR‐HA on COL II surface. d) Evaluation of the binding modes between COL II and WYR as determined by MD simulations. e) Representative plots of △*f* for the adsorption of DPPC liposomes on HA surface and HA on DPPC liposomes surface. f) The final adsorption mass of DPPC liposomes on HA surface and HA on DPPC liposomes surface. g) The schematic representation of DPPC liposomes on HA surface and HA complexed on DPPC liposomes surface and their interaction mechanism. h) The LSCM images of DPPC Liposomes into HMs. i) The bare mica surface morphology in water. j) The DPPC liposomes surface morphology on mica in water (the insert was the single liposome at 100 nm). k) The HAMA and DPPC liposomes mixtures surface morphology on mica in water.

To assess the ability of supramolecular lubricating hydrogels to target cartilage matrix, HA and WYR‐HA on COL II (the main component of damaged cartilage matrix) were monitored in situ by quartz crystal microbalance (QCM‐D) technique.^[^ ^18^ ^]^ **Figure** **4**a shows the frequency and dissipation responses of COL II biointerface with a concentration of 100 µg mL^−1^ in PBS over a bare Au surface at a rate of 50 µL min^−1^. When the baseline was stable followed by the injection of COL II solution, a decrease in frequency at ≈892 Hz and an increase in dissipation at ≈150 units were observed and the signal plateaus weren't up to a stable value in frequency after the 60 min, so we stopped the injection at this point and the surface was followed by rinsing with PBS. During the rinse, there was no apparent change in frequency and dissipation. Finally, we obtained COL II surface.

Figure 4b shows the QCM response signal in frequency and dissipation during the HA adsorbed on COL II biointerface, through the injection of an aqueous solution of HA in PBS over the bare Au chip. Upon initial injection of HA solution, a rapid decrease in frequency at ≈0.45 Hz and an increase in dissipation at ≈1.45 units were observed and the signal plateaus was up to a maximum adsorption value in frequency after the 15 min injection, with no further decrease but a increase when the flow of additional HA solution, suggesting the HA adsorption on COL II surface was saturated. At this point, the surface was followed by rinsing with PBS. During the rinse, there was a completely increase in frequency to 0 Hz. In contrast, upon initial injection of WYR‐HA solution on COL II surface (Figure 4c), a rapid decrease in frequency at ≈16.98 Hz and an increase in dissipation at ≈6.0 units were observed and the signal plateaus was up to a maximum adsorption value in frequency. At this point, the surface was followed by rinsing with PBS. During the rinse, there was a slightly increase in frequency to ‐11.97 Hz. This suggests that the adsorption of WYR‐HA on the surface of COL II was stable, possibly through specific recognition of the α1 chain of COL II,^[^ ^19^ ^]^ which is important for biolubricant to resist synovial fluid removal.

To further validate the high binding affinity between WYR‐HA and COL II, we conducted molecular dynamics (MD) simulations to examine the interactions between WYR and COL II. The resulting complex model of WYR with COL II is illustrated in Figure 4d. We calculated the variations in contact area and hydrogen bond numbers formed between WYR and COL II, as depicted in Figure S5a (Supporting Information). Analyzing the data from the final 10 nanos of the simulation, we determined that the average hydrogen bond numbers between the two components was 1.2. Additionally, we computed the free energy of binding (ΔBind) for the interacting components, along with the contributions from van der Waals interactions (ΔEvdW), electrostatic interactions (ΔEele), polar solvation free energy (ΔGpb), and nonpolar solvation free energy (ΔGsa), as shown in Figure S5b (Supporting Information). The values obtained were ΔEvdW = −31.84 kcal mol^−1^, ΔEele = −164.45 kcal mol^−1^, ΔGpb = 181.79 kcal mol^−1^, and ΔGsa = −3.37 kcal mol^−1^ for the interaction between WYR and COL II. These results indicate that the primary interactions between WYR and COL II are mediated by electrostatic and van der Waals forces. Furthermore, the average ΔBind value of −15.23 kcal mol^−1^ suggests a stable binding affinity of WYR‐HA to the surface of COL II, which is advantageous for achieving prolonged lubrication properties.

### 2.4 Assembly Behavior of Supramolecular Lubricating Hydrogel Microspheres

To assess the efficacy of supramolecular hydrogel assembly, we investigated the irreversible adsorption capacity between DPPC liposomes and HA molecules utilizing QCM‐D. The curves and schematic representations of DPPC liposomes on the HA surface, as well as HA on the DPPC liposomes surface, are illustrated in Figure 4e–g. The adsorption response signal exhibited a decrease when the DPPC solution was introduced to the HA surface; however, the frequency value remained relatively stable following subsequent rinsing with PBS. This observation indicates that DPPC liposomes stably assembled and adsorbed onto the HA surface, culminating in a final frequency change (∆*f*) of 13.92 Hz. Upon the introduction of HA solution, a frequency alteration of ≈17.85 Hz was recorded. Nevertheless, after rinsing with PBS, no significant change in frequency was noted, suggesting a stable adsorption of HA onto the DPPC liposomes surface. This stability may be attributed to the integration of various intermolecular electrostatic interactions, including ion‐dipole interactions between HA and DPPC liposomes. The final adsorption quantities of DPPC liposomes on the HA surface and HA on the DPPC liposomes surface were measured at 246  and 316 ng cm^−2^, respectively.

Furthermore, HMs incorporating DiI‐labeled DPPC liposomes were analyzed through laser scanning confocal microscopy (LSCM) to verify the successful formation of the DPPC liposome complex. As illustrated in Figure 4h, the red fluorescence exhibited a uniform distribution across the HMs, thereby confirming the effective incorporation of the DPPC liposomes. Figure 4i–k present bare mica surfaces that were initially incubated with either DPPC liposomes or a DPPC+HAMA mixture, followed by rinsing. The intact liposomes were observed as single entities dispersed across the mica surface. In contrast, mica surfaces treated with the DPPC+HAMA mixture and subsequently rinsed displayed visibly close‐packed layers and the formation of HA/phospholipid complexes on the surfaces, suggesting that HAMA in the bulk solution interacted with the liposomes adhered to the surface. This finding implies that hydrogel microspheres may provide sustained supramolecular lubrication for the damaged matrix interface.

### 2.5 Cyto‐Compatibility of Supramolecular Lubricating Hydrogel Microspheres

**Figure 5:**
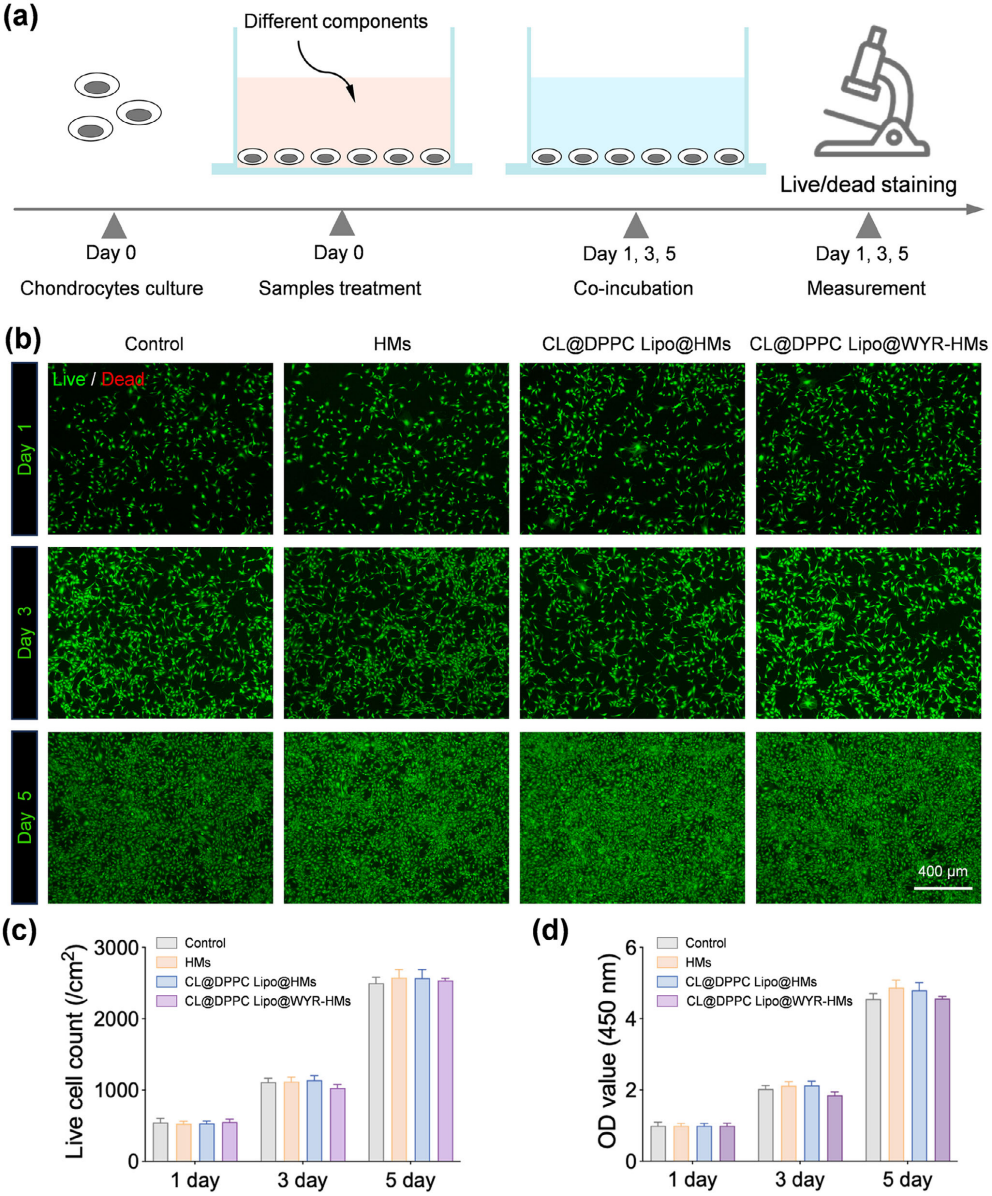
In vitro biocompatibility of CL@DPPC Lipo@WYR‐HMs. a) Co‐incubation to evaluate the biocompatibility of control, HMs, CL@DPPC Lipo@HMs and CL@DPPC Lipo@WYR‐HMs. b) Live/Dead results of the control, HMs, CL@DPPC Lipo@HMs and CL@DPPC Lipo@WYR‐HMs. c) Viable cell count of the control, HMs, CL@DPPC Lipo@HMs and CL@DPPC Lipo@WYR‐HMs. d) The CCK‐8 results assay showed the cytotoxicity of control, HMs, CL@DPPC Lipo@HMs and CL@DPPC Lipo@WYR‐HMs on chondrocyte. [Correction added on 04 September 2025 after online publication: Figure 5 is updated in this version.]

As a biolubricant, CL@DPPC Lipo/WYR‐HMs are expected to demonstrate cyto‐compatibility. To assess the effects of CL@DPPC Lipo/WYR‐HMs on the viability and proliferation of chondrocytes, both a Live/Dead assay and a cell counting kit‐8 (CCK‐8) assay were performed (**Figure** **5**a). The findings illustrated in Figure 5b indicate that nearly all cells survived the 5‐day incubation period, with no statistically significant differences detected among the various groups. Consistent with the results of the Live/Dead assay, the CCK‐8 assay revealed a progressive increase in cell counts across all groups over time, again showing no significant differences between them (Figure 5c,d). These findings imply that CL@DPPC Lipo/WYR‐HMs exhibit favorable cyto‐compatibility.

### 2.6 In Vivo Therapeutic Effect of Supramolecular Lubricating Hydrogel Microspheres

**Figure 6:**
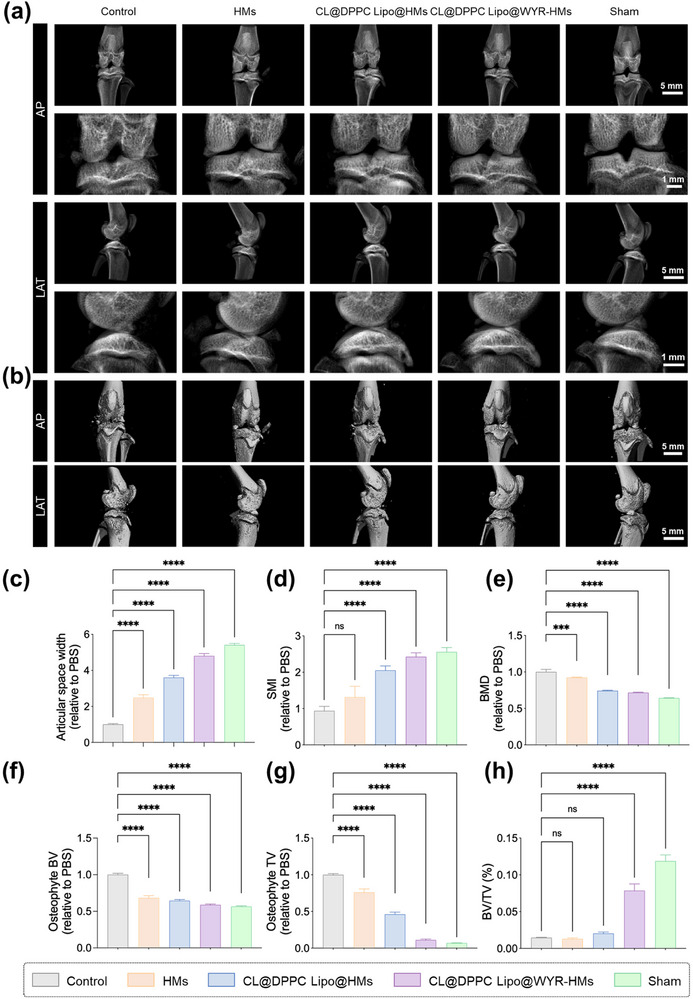
Micro‐CT evaluation. a) The measured X‐ray images of the obtained knee joint. b) The micro‐CT images of the obtained knee joint. c) The relative JSW. d) The measured relative structure model index (SMI). e) The relative osteophyte volume (BMD). f) The relative tissue volume (TV). g) The relative bone volume (BV). h) The relative BV/TV. (****p < 0.0001).

This investigation employed a rat OA model to assess the efficacy of the supramolecular lubricating hydrogel microspheres in reducing joint wear and osteoarthritic degeneration.^[^ ^20^ ^]^ OA is characterized by cartilage loss and damage,^[^ ^21^ ^]^ which typically manifests as a reduction in joint space as observed on X‐ray imaging.^[^ ^22^ ^]^ As illustrated in **Figure** **6**a,c, the joint space widths (JSWs) were significantly diminished in the control, HMs, CL@DPPC Lipo@HMs, and CL@DPPC Lipo@WYR‐HMs groups when compared to the sham group. Conversely, the CL@DPPC Lipo@WYR‐HMs group exhibited a notable increase in JSWs relative to the control group. This finding suggests that CL@DPPC Lipo@WYR‐HMs may function as a bio‐lubricant capable of mitigating cartilage degradation. Osteophyte formation, a characteristic feature of OA that serves as a compensatory response to joint damage,^[^ ^23^ ^]^ was observed in the control, HMs, CL@DPPC Lipo@HMs, and CL@DPPC Lipo@WYR‐HMs groups, but was absent in the sham group, as depicted in Figure 6b. Furthermore, the structural metrics including SMI, BMD, TV, BV, and BV/TV were more comparable in the sham group than in the control group (Figure 6d–h), indicating that CL@DPPC Lipo@WYR‐HMs may serve as a biolubricant to alleviate joint damage and diminish osteophyte formation.

**Figure 7:**
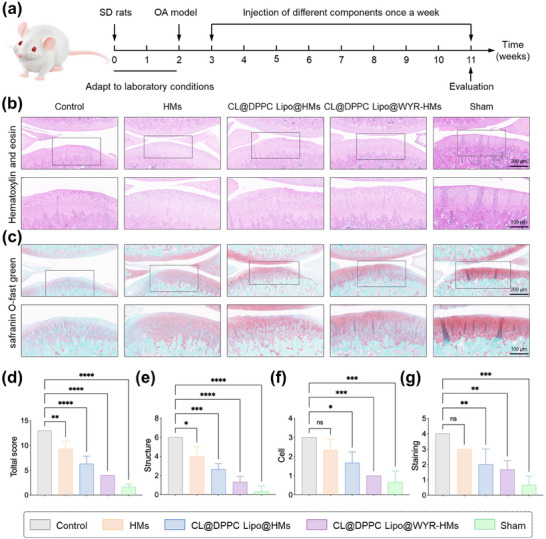
Histological staining. a) The OA model and samples treatment. b) Representative images of HE staining in control, HMs, CL@DPPC Lipo@HMs, CL@DPPC Lipo@WYR‐HMs and sham groups. c) Safranin O‐fast green staining in control, HMs, CL@DPPC Lipo@HMs, CL@DPPC Lipo@WYR‐HMs and sham groups. d) Total Mankin scores of articular cartilage in control, HMs, CL@DPPC Lipo@HMs, CL@DPPC Lipo@WYR‐HMs and sham groups. Mankin score presented as e) cartilage structure, f) cellular abnormalities, and (g) matrix staining. (*p < 0.5, **p < 0.01, ***p < 0.001, ****p < 0.0001).

In addition to radiological assessments, we utilized hematoxylin‐eosin (HE) and Safranin O‐fast green staining techniques to evaluate histological changes in the cartilage.^[^ ^24^ ^]^ As illustrated in **Figure** **7**a–c, the cartilage in the sham group exhibited a smooth surface,^[^ ^25^ ^]^ typical structural organization,^[^ ^26^ ^]^ normal cellularity,^[^ ^27^ ^]^ and a high intensity of Safranin O‐fast green staining.^[^ ^28^ ^]^ Conversely, the articular cartilage in the control group displayed significant surface erosion, disorganized chondrocytes, and diminished Safranin O‐fast green staining intensity. Notably, the CL@DPPC Lipo@WYR‐HMs group did not present any substantial degenerative alterations, a finding that was corroborated by the Mankin score (Figure 7d). While the articular cartilage in the CL@DPPC Lipo@WYR‐HMs group showed some degenerative changes relative to the sham group, it maintained a relatively intact and organized cartilage structure when compared to the control group, as evidenced by the Mankin scores (total, structural, cellular, and strain in Figure 7d–g). These results further substantiate the efficacy of supramolecular lubrication provided by CL@DPPC Lipo@WYR‐HMs.

**Figure 8:**
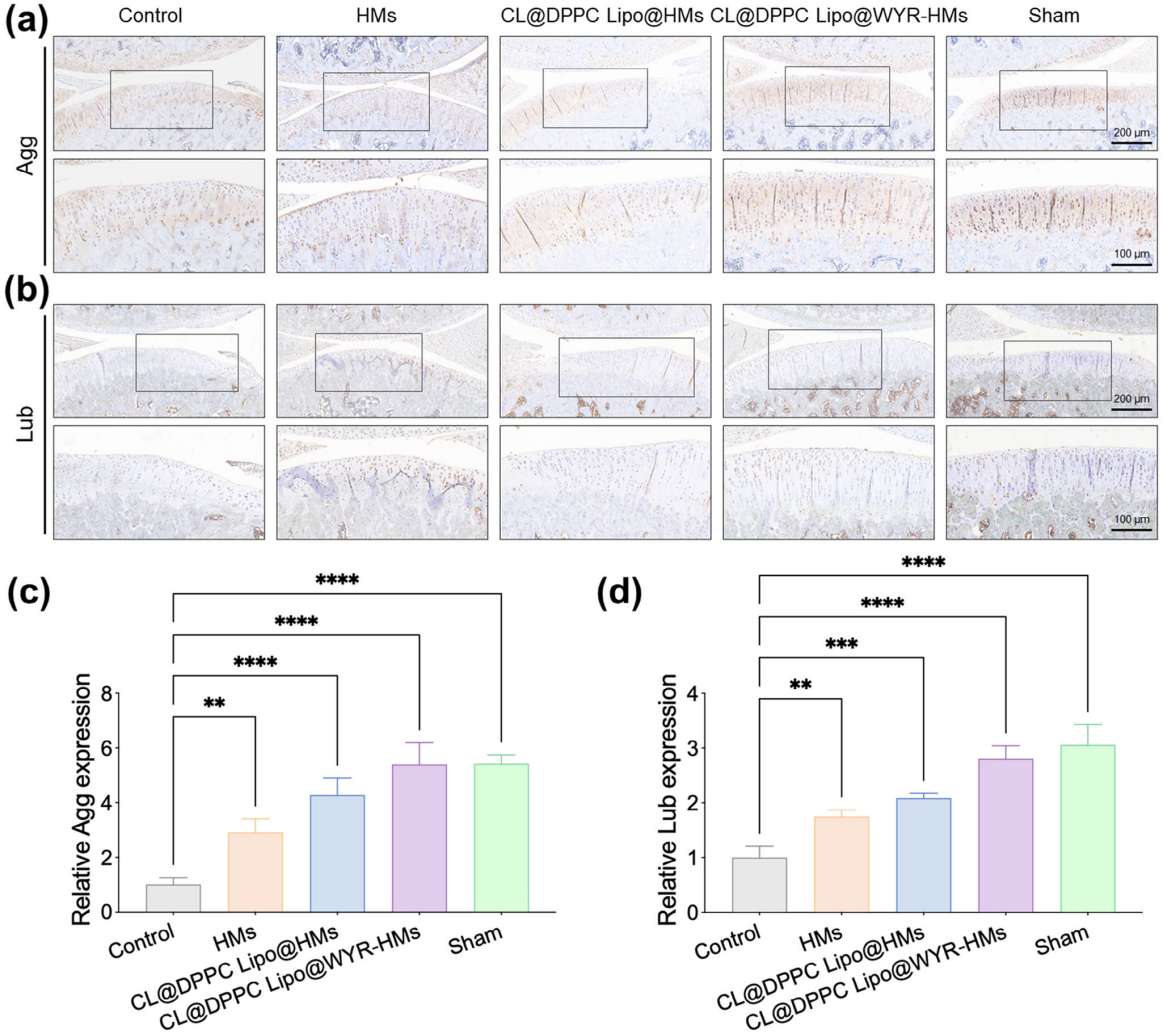
Immunohistochemistry staining. a) The images of Agg immunohistochemical staining. b) The images of Lub immunohistochemical staining. c) Relative Agg expression. d) Relative Lub expression. (**p < 0.01, ***p < 0.001, ****p < 0.0001).

**Figure 9:**
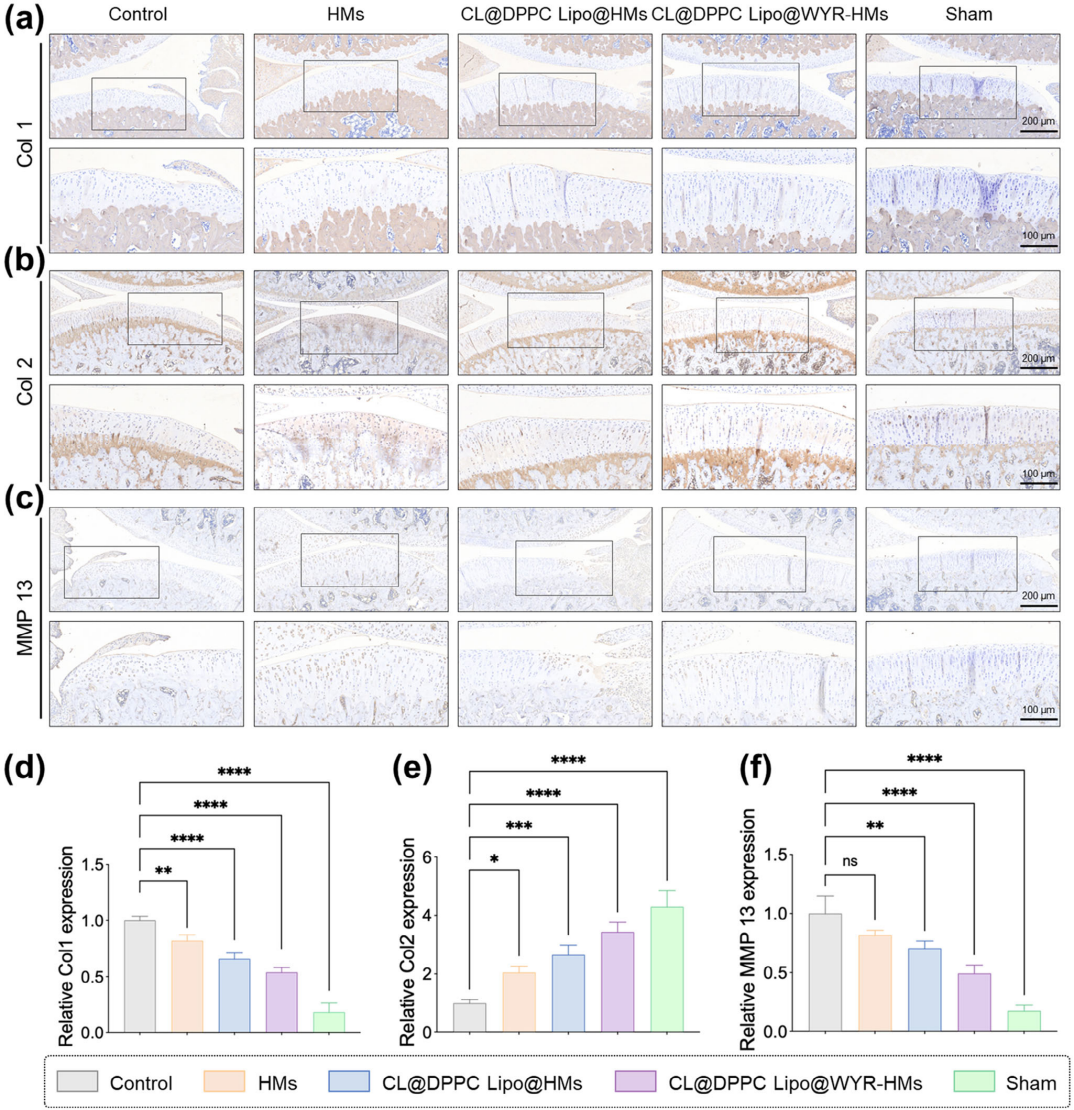
Immunohistochemistry staining. a) The Col 1 immunohistochemical staining images. b) The Col 2 immunohistochemical staining images. c) The MMP 13 immunohistochemical staining images. d) Relative Col 1 expression. e) Relative Col 2 expression. f) Relative MMP 13 expression. (*p < 0.5, **p < 0.01, ***p < 0.001, ****p < 0.0001).

Further, we evaluated the secretion level of supramolecular lubricating hydrogel microspheres to promote lubricating functional factors in damaged cartilage interface. As shown in **Figure** **8**a–d, the expression of lubricin (Lub) and aggrecan (Agg) was significantly increased in the microsphere group compared with the control group, with higher expression in the CL@DPPC Lipo@WYR‐HMs group. This suggests that supramolecular lubricating hydrogel microspheres can provide a lubricating mechanical microenvironment at damaged matrix interface, which in turn maintains cellular catabolic homeostasis to slow OA progression. The loss of lubrication in tissue injury areas can lead to fibrotic tissue formation. Eventually, the proliferating chondrocytes transform into a fibroblast‐like phenotype, inducing cartilage degradation and dedifferentiation.^[^ ^29^ ^]^ Therefore, we employed immunohistochemistry to assess the influence of supramolecular lubricating hydrogel microspheres on the expression of ECM synthesis. **Figure** **9**a–f shows the expression levels of inflammatory factor MMP 13, and ECM proteins Col 1, Col 2 in the control, HMs, CL@DPPC Lipo@HMs, CL@DPPC Lipo@WYR‐HMs and sham groups at 8 weeks post‐surgery. Compared to the control group, all microsphere groups (HMs, CL@DPPC Lipo@HMs, CL@DPPC Lipo@WYR‐HMs) exhibited significantly reduced MMP 13 and Col 1 expression, while Col 2 expression was markedly increased. The CL@DPPC Lipo@WYR‐HMs group consistently demonstrated the most favorable expression profile among all groups. These findings suggested that supramolecular lubricating hydrogel microspheres, particularly the CL@DPPC Lipo@WYR‐HMs formulation, effectively inhibited MMP13 activity via CL, fibrotic cartilage tissue formation, and promote extracellular matrix synthesis in damaged cartilage.

## 3 Conclusion

In this study, we present a supramolecular lubricating hydrogel microsphere supplementation formulation. This hydrogel microspheres could provide stable mechanophysical protection to cells at damaged matrix through interfacial hydration lubrication. In vitro experiments, supramolecular lubricating hydrogel microspheres could autonomously regulate lubrication structure: in the early and middle stages, microspheres could actively provide microscale supramolecular lubrication (µ ≈ 0.04) for damaged matrix; in the late stage, the wear debris induced by endogenous enzymatic decomposition transmitted to provide nanoscale supramolecular lubrication for damaged matrix (µ ≈ 0.06). In vivo experiments, supramolecular lubricating hydrogel microspheres stimulated the efficient synthesis of extracellular matrix and autonomous secretion of lubrication factors at the cellular level. This supramolecular lubricating hydrogel would also provide a potential therapeutic approach for other biological tissues or interfaces with lubrication problems.

## 4 Experimental Section

### Preparation and Characterization of WYR‐HA

The carboxyl groups on HA (Bloomage Biotechnology Corporation Limited) (2.5 mm) were activated by EDC (Shanghai aladinin) (2.5 mm) in 10 mm PBS buffer at pH 5.5 for 15 min. Next, 2.5 mM WYR (GenScript) containing amino groups was added and reacted with activated carboxyl groups for 2 h under stirring at room temperature. Upon the reaction completion, solution was dialyzed by deionized water (pH 5.5, adjusted with 1 M HCl) using a dialysis bag (MWCO≈8000‐14 000 Da) for 3 days, and then lyophilized to obtain the final purified product, named as WYR‐HA.

### Preparation and Characterization of WYR‐HAMA

WYR‐HA was subjected to a reaction with MA in a 2% (w/w) solution of deionized water at a pH of 8.0. The mixture was stirred in an ice bath for 24 h, followed by purification through dialysis in deionized water with a molecular weight cutoff of ≈8000–14 000 Da for three days. The product was then lyophilized for an additional three days, yielding a white powder designated as WYR‐HAMA. The methacrylation degree was dissolved in heavy water and evaluated by 400 MHz 1H NMR (Bruker, Germany).

### Preparation and Characterization of DPPC Liposomes

DPPC, cholesterol, octadecylamine, and inhibitor CL‐82198 were dissolved together in 3 mL of chloroform and evaporated to form a thin membrane under reduced pressure, followed by hydrating it in a sample vial with 2 mL of deionized water under sonication and a liposome extruder.^[^ ^10^ ^]^ Replenish the liposomes with deionized water to 5 mL, and 10 uL of DPPC liposome solution with deionized water was measured particle size and potential by Nano particle size and Zeta potential analyzer and repeated by 3 times.

### Preparation and Characterization of Supramolecular Lubricating Hydrogel Microspheres

A mixture consisting of 2 wt.% HAMA, 1 wt.% DPPC liposome, and 0.2 wt.% photoinitiator was employed to generate pre‐gel droplets (in situ generation by microfluidics in oil‐water phase shear) within a oil composed of 95 wt.% paraffinic acid oil (Shanghai aladinin) and 5 wt.% Spectra 80 (Shanghai aladinin) (This ratio optimizes the shear capacity of the oil phase in the aqueous phase). The resulting droplets were subsequently subjected to crosslinking through UV irradiation for 10 min. Following this process, the crosslinked supramolecular lubricating hydrogel microspheres underwent a series of washings with ether (removal of the oil from the microsphere) and deionized water, after which they were preserved in deionized water for prospective applications. The surface morphology of supramolecular lubricating hydrogel microspheres was analyzed using a SEM (ZEISS, Germany). To verify the successful incorporation of liposomes into the microspheres, additional surface EDS analyses were performed.

### Lubrication Measurements of Supramolecular Lubricating Hydrogel Microspheres

The lubrication properties of supramolecular lubricating hydrogels were assessed through tribological testing conducted using a universal materials tester (Bruker, Germany) in a linear reciprocating mode at ambient temperature. The tribological experiments employed a pin‐on‐disk configuration, with a polytetrafluoroethylene pin (contact surface diameter: 5 mm) serving as the upper specimen and GCr15 steel balls (diameter: 9 mm) functioning as the lower specimen. Prior to each test, 15 mL of hydrogels, specifically CL@DPPC Lipo@HMs and CL@DPPC Lipo@WYR‐HMs, at a concentration of 10 mg mL^−1^, were introduced into the contact area as the lubricant. The parameters for the oscillation amplitude, frequency, applied load, and duration of the test were set at 4 mm, 1 Hz, 1 N (with additional tests at 5 and 10 N), and 600 s, respectively.

### Degradation Test

An analysis of the degradation of supramolecular lubricating hydrogel microspheres was conducted through enzymatic degradation experiments. The microspheres were placed in phosphate‐buffered saline (PBS, pH 7.4) with hyaluronidase (1000 U mL^−1^). Specifically, 1 mL samples of the microsphere suspensions were kept in a shaking incubator at 37 °C with continuous agitation at 80 rpm. To ensure the enzymatic activity was maintained throughout the study, the hyaluronidase solution was refreshed with new enzyme solution every 48 h. The morphological changes were carefully observed and recorded using optical microscopy.

### Supramolecular Lubricating Hydrogel Microspheres to Target Damaged Matrix and Assembly Behavior

Adsorption of WYR‐HA or HA on COL II surfaces, DPPC on HA surface and HA on DPPC liposome surface can be used QCM‐D to test the adsorbed amount by the change in Δ*f* and dissipation. The relationship between the change in mass (Δ*m*) and Δ*f* is expressed by the Sauerbrey relation: Δ*m* = $\frac{\rho_{q}h_{q}}{nf_{0}}\Delta f=-C\Delta f$, *C* = 17.8 ng cm^−2^ Hz^−1^.^[^ ^30^ ^]^

COL II was dissolved to 100 µg mL^−1^, and the COL II surface was prepared as follows: (i) PBS was introduced into the QCM‐D over the Au surface until a stable baseline was observed; (ii) COL II solution was subsequently injected into the flow cell for 60 min; and (iii) the chips were rinsed with the PBS solution to achieve a new plateau in the signal response. HA and WYR‐HA were dissolved in PBS. Following on from step (iii) above (iv) HA or WYR‐HA solution was injected into the flow cell; and (v) the chips were rinsed with PBS solution to achieve a new plateau in the signal response. Molecular dynamics simulations between COL II and WYR were also performed using the GROMACS. The simulation methodology and procedure were seen in previous work.^[^ ^9^ ^]^

In a similar manner, thioled HA (tHA) (note: HA can't adsorb on the gold surfaces, so we use tHA to chemabsorb on the gold surfaces for the measurement) was solubilized in PBS, and the gold surfaces were modified by incubating them in the tHA solution for 1 h. Following the procedures outlined in step (i), a solution of DPPC liposomes was introduced into the flow cell as described in step (iii). Subsequently, a solution of HA was injected into the flow cell, and the chips were rinsed with PBS to establish a new plateau in the signal response. To confirm the successful incorporation of DPPC liposomes within HMs, the liposomes were initially labeled with DiI and then combined with HMs. The DiI‐labeled liposomes embedded within HMs were subsequently visualized using a LSCM. Additionally, imaging of mica, DPPC liposomes, and the DPPC liposome/HAMA mixture on a mica substrate was conducted using a FastScan Bio instrument to elucidate the assembly behavior.

### Biocompatibility of Supramolecular Lubricating Hydrogel Microspheres

To assess the cytocompatibility of the CL@DPPC Lipo/WYR‐HMs, chondrocytes were cultured in 24‐well plates at a density of 20 000 cells per well. The viability of the cells was evaluated through a Live/Dead staining assay following co‐culturing periods of 1, 3, and 5 days, while cell proliferation was quantified using a CCK‐8 assay. In Live/Dead staining assay, cells were treated with calcein‐AM and propidium iodide for a duration of 30 min, after which they were examined using a fluorescence microscope. For the CCK‐8 assay, CCK‐8 solution was added to culture medium, and the absorbance at 450 nm was measured two h later.

### In Vivo Experiments with Supramolecular Lubricating Hydrogel Microspheres

The animal experiments performed adhered to the principles of animal welfare and ethics, receiving approval from the Ethics Committee of the First Affiliated Hospital of Chongqing Medical University (Approval Number: IACUC‐CQMU‐2023‐0108). Specially, a total of 20 male Sprague‐Dawley (SD) rats, aged 12 weeks, were randomly assigned to either the sham group (4 rats) or the OA group (16 rats). In OA group, following anesthesia, the skin on the operated side was prepared and disinfected. A small incision was made on the medial aspect of the knee joint, allowing for layer‐by‐layer exposure of the joint cavity, during which the medial meniscus was resected. The joint cavity was subsequently closed in layers and sutured. The sham group underwent the same surgical procedure as the OA group, with the exception of the medial meniscectomy. One week post‐surgery, the OA model group was further subdivided into four subgroups (4 rats each), with the joint cavity receiving weekly injections of PBS, HMs, CL@DPPC Lipo/HMs, and CL@DPPC Lipo/WYR‐HMs formulations, respectively. Eight weeks post‐surgery, the knee joints were harvested and subjected to X‐ray examination and micro‐CT analysis.

The isolated knees were fixed in paraformaldehyde, decalcified, paraffin‐embedded, and sectioned in the sagittal plane. The sections were stained with HE and Safranin O‐fast green for histological assessment. Two blinded observers evaluated the pathology of the knee joints using a modified Mankin scoring system. And the sections were incubated overnight at 4 °C with a primary antibody, followed by a 1 h incubation with a secondary antibody. Subsequently, the paraffin sections were stained using a 3,3′‐diaminobenzidine substrate. Image J software was employed to quantify the expression levels.

### Statistical Analysis

All data are presented as mean ± standard deviation. Statistical analyses were performed using a Student's *t*‐test and one‐way analysis of variance (ANOVA) via prism 9 softwire. A p‐value of less than 0.05 was considered statistically significant for all comparisons.

## Conflict of Interest

The authors declare no conflict of interest.

## Supporting information



Supporting Information

## Data Availability

The data that support the findings of this study are available from the corresponding author upon reasonable request.

## References

[1] a) J. Seror , L. Zhu , R. Goldberg , A. J. Day , J. Klein , Nat. Commun. 2015, 6, 6497; 25754223 10.1038/ncomms7497 PMC4366511

[2] a) C. D. DeMoya , A. Joenathan , T. B. Lawson , D. T. Felson , T. P. Schaer , M. Bais , M. B. Albro , J. Mäkelä , B. D. Snyder , M. W. Grinstaff , Nat. Rev. Rheumatol. 2024, 20, 432; 38858605 10.1038/s41584-024-01125-5 PMC11348290

[3] M.‐H. Bai , B. Zhao , Z.‐Y.‐T. Liu , Z.‐L. Zheng , X. Wei , L. Li , K. Li , X. Song , J.‐Z. Xu , Z.‐M. Li , Adv. Mater. 2022, 34, 2108848. 10.1002/adma.202108848 35075678

[4] X. Zhang , J. Wang , H. Jin , S. Wang , W. Song , J. Am. Chem. Soc. 2018, 140, 3186. 29380600 10.1021/jacs.7b12886

[5] Y. Wang , Y. Sun , A.‐J. Avestro , P. R. McGonigal , H. Zhang , Chem 2022, 8, 480.

[6] a) H. Qiu , J. Deng , R. Wei , X. Wu , S. Chen , Y. Yang , C. Gong , L. Cui , Z. Si , Y. Zhu , R. Wang , D. Xiong , Int. J. Biol. Macromol. 2023, 243, 125249; 37295698 10.1016/j.ijbiomac.2023.125249

[7] a) J. Clarke , Nat. Rev. Rheumatol. 2021, 17, 707; 10.1038/s41584-021-00712-0 34675382

[8] a) N. Das , L. G. N. de Almeida , A. Derakhshani , D. Young , K. Mehdinejadiani , P. Salo , A. Rezansoff , G. D. Jay , C. P. Sommerhoff , T. A. Schmidt , R. Krawetz , A. Dufour , Nat. Commun. 2023, 14, 1910; 37024468 10.1038/s41467-023-37598-3 PMC10079686

[9] H. Yuan , P. Xiao , B. Sarmento , G. Chen , W. Cui , Adv. Funct. Mater. 2024, 34, 2406668.

[10] Y. Lei , Y. Wang , J. Shen , Z. Cai , C. Zhao , H. Chen , X. Luo , N. Hu , W. Cui , W. Huang , Sci. Adv. 8, abl6449. 10.1126/sciadv.abl6449 PMC8809544 35108047

[11] S. Amorim , C. A. Reis , R. L. Reis , R. A. Pires , Trends Biotechnol. 2021, 39, 90. 32654775 10.1016/j.tibtech.2020.06.003

[12] H. Yuan , P. Xiao , B. Sarmento , G. Chen , W. Cui , Adv. Funct. Mater. 2024, 34, 2406668.

[13] a) A. Dėdinaitė , D. C. F. Wieland , P. Bełdowski , P. M. Claesson , Adv. Colloid Interface Sci. 2019, 274, 102050; 31669714 10.1016/j.cis.2019.102050

[14] E. Hermans , J. Vermant , Soft Matter 2014, 10, 175. 24651838 10.1039/c3sm52091a

[15] a) X. Han , F. Wang , J. Shen , S. Chen , P. Xiao , Y. Zhu , W. Yi , Z. Zhao , Z. Cai , W. Cui , D. Bai , Adv. Mater. 2024, 36, 2306993; 10.1002/adma.202306993 37851922

[16] Y. Lei , X. Wang , J. Liao , J. Shen , Y. Li , Z. Cai , N. Hu , X. Luo , W. Cui , W. Huang , Bioact. Mater. 2022, 16, 472. 35415286 10.1016/j.bioactmat.2022.02.016 PMC8967971

[17] a) P. S. Theobald , D. Dowson , I. M. Khan , M. D. Jones , J. Biomech. 2012, 45, 1972; 22704825 10.1016/j.jbiomech.2012.05.005

[18] a) Y. Ji , Z.‐W. Yin , Z. Yang , Y.‐P. Deng , H. Chen , C. Lin , L. Yang , K. Yang , M. Zhang , Q. Xiao , J.‐T. Li , Z. Chen , S.‐G. Sun , F. Pan , Chem. Soc. Rev. 2021, 50, 10743; 34605826 10.1039/d1cs00629k

[19] A. Singh , M. Corvelli , S. A. Unterman , K. A. Wepasnick , P. McDonnell , J. H. Elisseeff , Nat. Mater. 2014, 13, 988. 25087069 10.1038/nmat4048 PMC6317357

[20] P. Xiao , X. Han , Y. Huang , J. Yang , L. Chen , Z. Cai , N. Hu , W. Cui , W. Huang , Bioact. Mater. 2024, 32, 242. 37869722 10.1016/j.bioactmat.2023.09.010 PMC10589729

[21] J. N. Katz , K. R. Arant , R. F. Loeser , J. Am. Med. Assoc. 2021, 325, 568.

[22] E. H. Park , J. Fritz , Best Pract. Res. Clin. Rheumatol. 2023, 37, 101866. 37659890 10.1016/j.berh.2023.101866

[23] T. Fan , G. Ruan , B. Antony , P. Cao , J. Li , W. Han , Y. Li , S. N. Yung , A. E. Wluka , T. Winzenberg , F. Cicuttini , C. Ding , Z. Zhu , Osteoarthritis Cartilage 2021, 29, 1296. 34216729 10.1016/j.joca.2021.06.008

[24] S.‐z. Jing , S.‐h. Yang , Y.‐k. Qu , H.‐h. Hao , H. Wu , Curr. Med. Sci. 2024, 44, 355. 38570439 10.1007/s11596-024-2854-6

[25] J. Chen , S. Liu , Y. Li , S. Zhang , X. Li , J. Wang , Chem. Eng. J. 2023, 473, 145180.

[26] L. Liu , Y. Xian , W. Wang , L. Huang , J. Fan , W. Ma , Y. Li , H. Liu , J.‐K. Yu , D. Wu , ACS Nano 2023, 17, 24308. 37975685 10.1021/acsnano.3c10139

[27] J. Deng , R. Wei , H. Qiu , X. Wu , Y. Yang , Z. Huang , J. Miao , A. Liu , H. Chai , X. Cen , R. Wang , Carbohydr. Polym. 2024, 330, 121821. 38368102 10.1016/j.carbpol.2024.121821

[28] C. Xie , Q. Sun , Y. Dong , H. Lu , W. Li , Z. Lin , K. Li , J. Cheng , Z. Liu , J. Qi , B. Tang , L. Lin , ACS Nano 2023, 17, 12842. 37326369 10.1021/acsnano.3c04241

[29] a) J. Li , H. Jiang , Z. Lv , Z. Sun , C. Cheng , G. Tan , M. Wang , A. Liu , H. Sun , H. Guo , F. Chen , Z. Liu , Y. Fei , Y. Liu , R. Wu , X. Xu , W. Yan , Q. Jiang , D. Shi , A. stabilization , Sci. Adv. 8, abn8420; 10.1126/sciadv.abn8420 PMC9674280 36399569

[30] a) G. Rudolph , A. Hermansson , A.‐S. Jönsson , F. Lipnizki , Sep. Purif. Technol. 2021, 254, 117578;

