# Supplementary material for: Supramolecular Lubricating Hydrogel Microspheres Reshape Damaged Matrix Regeneration
Source: Adv Sci (Weinh). 2025 Jul 16;12(37):e04319. doi: 10.1002/advs.202504319 (PMC12499493; doi:10.1002/advs.202504319)
Supplement: Supplementary file 1 — Supporting Information [file ADVS-12-e04319-s001.docx]

Supporting Information

Supramolecular Lubricating Hydrogel Microspheres Reshape Damaged Matrix Regeneration

Hui Yuan, Pengcheng Xiao, Wei Huang, Wenguo Cui*


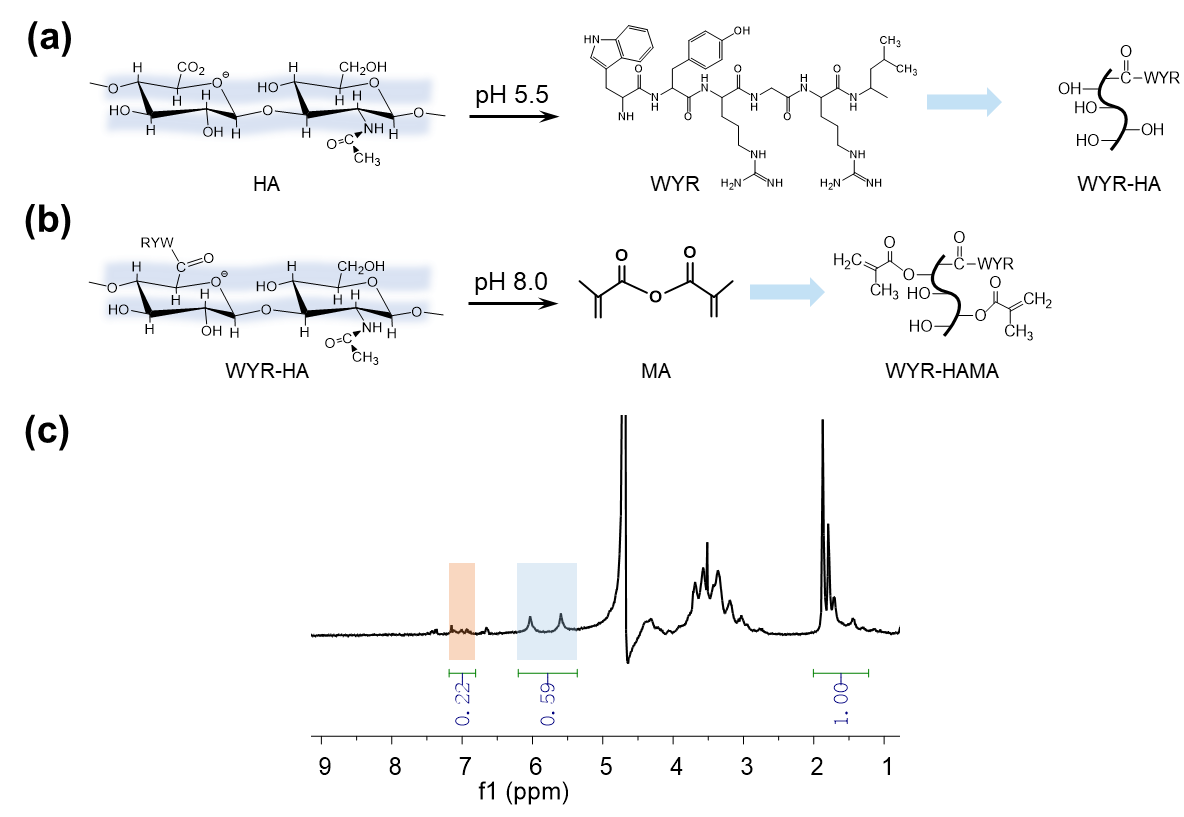


**Figure S1.** (a) The chemical structures of WYR-HA. (b) The chemical structures of WYR-HAMA. (c) 1H NMR spectra of WYR-HAMA.


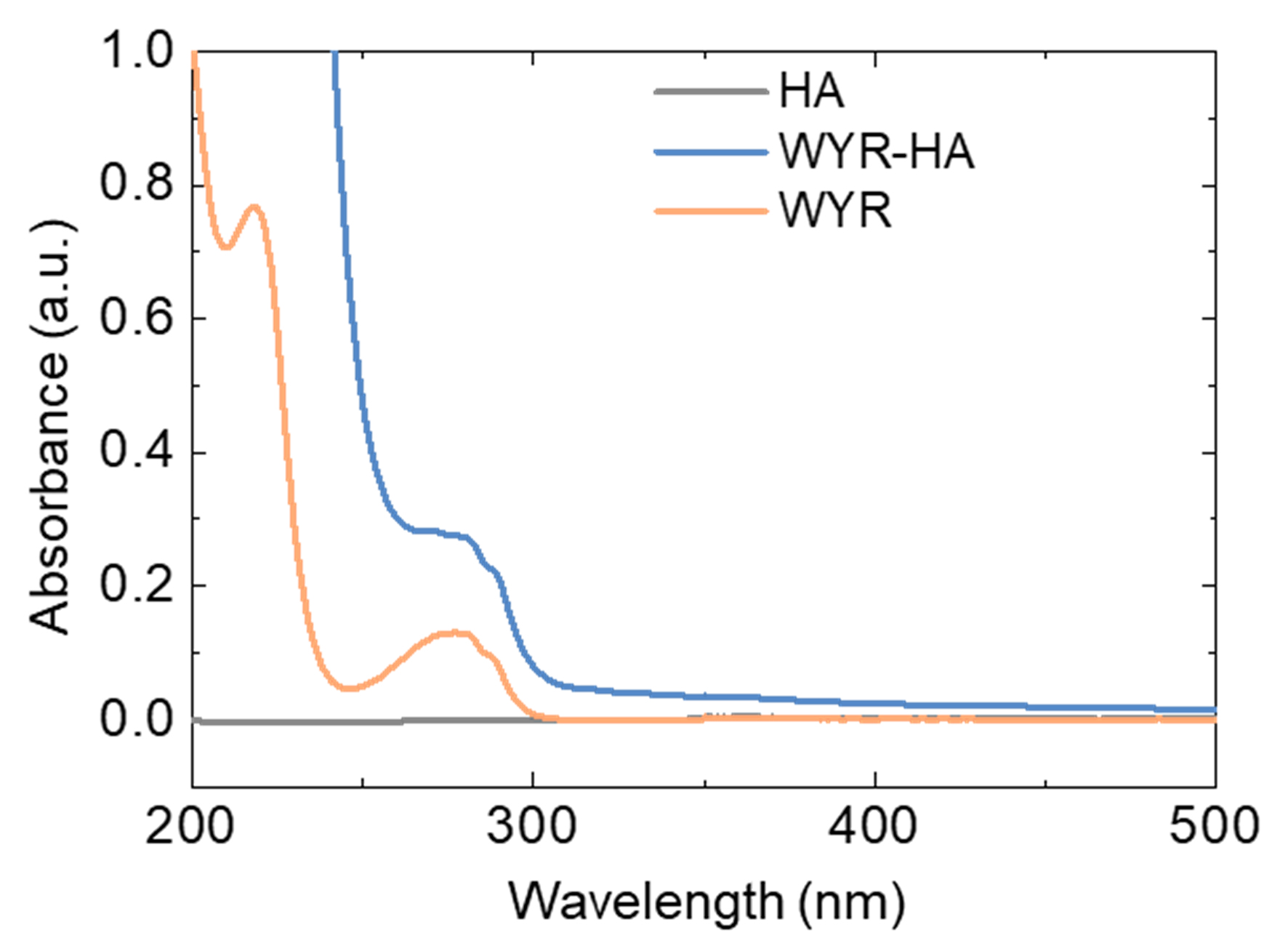


**Figure S2.** The UV-vis spectra of HA, WYR, and HA-WYR conjugate.


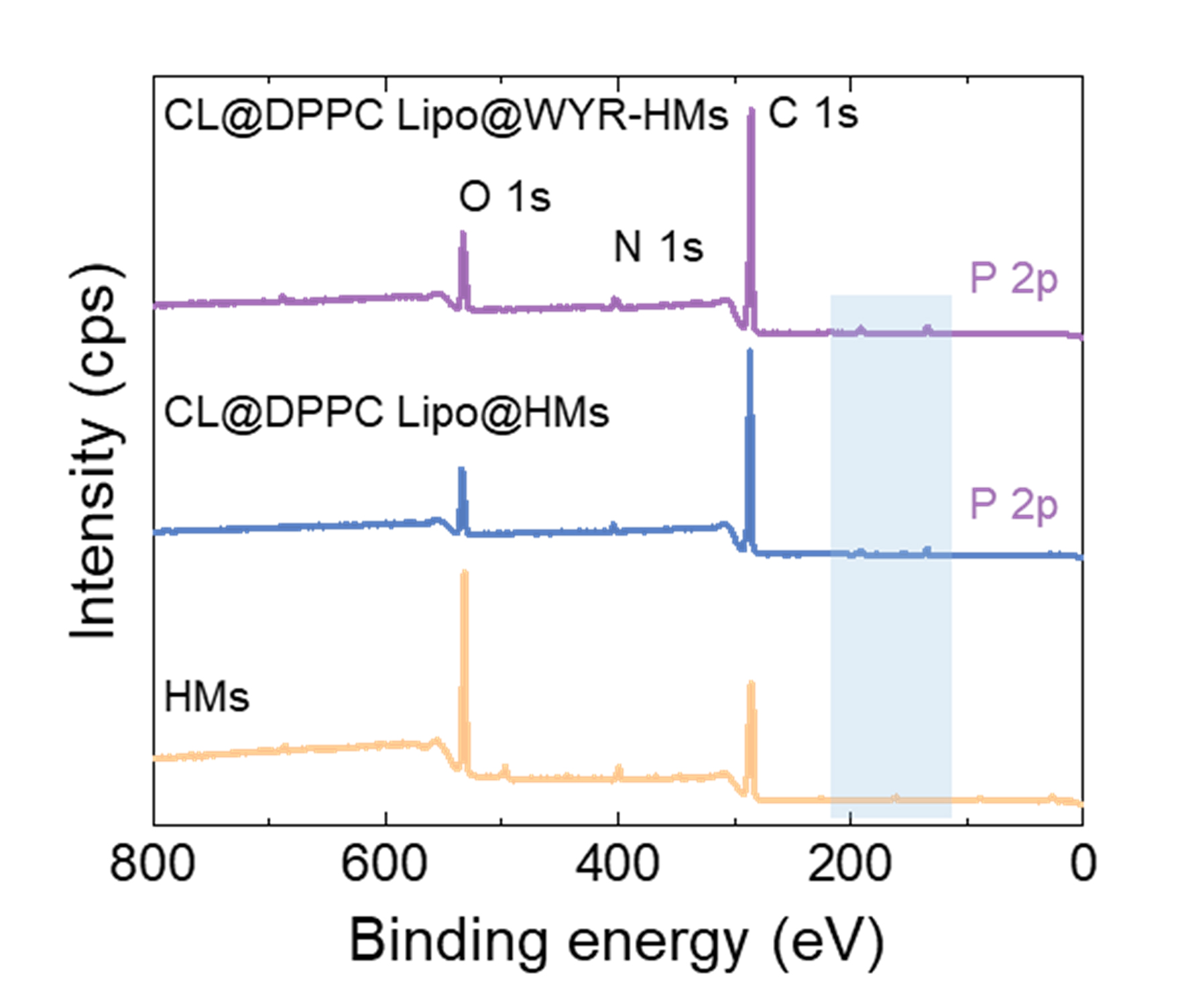


**Figure S3.** XPS analysis of HMs, CL@DPPC Lipo/HMs, CL@DPPC Lipo/WYR-HMs.


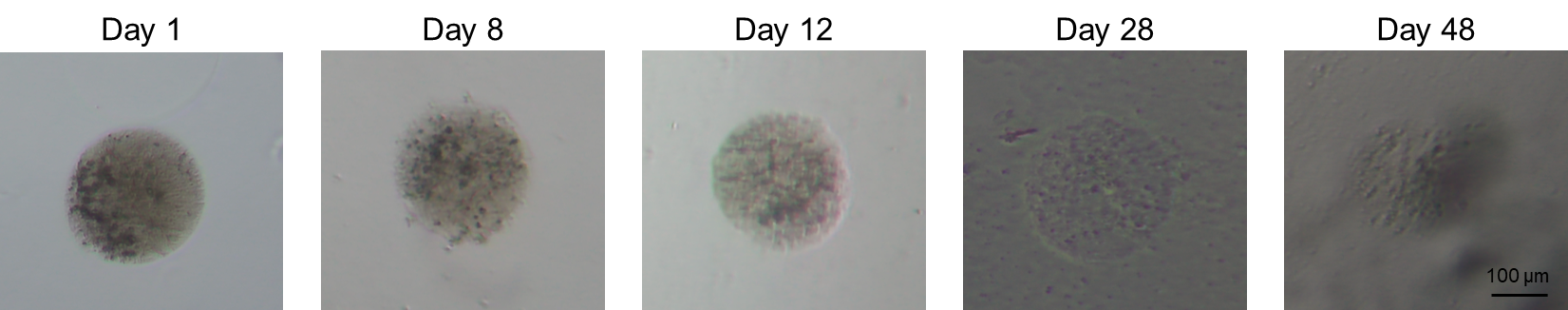


**Figure S4.** Morphological changes of the supramolecular lubricating hydrogel microspheres in day 1, 8, 12, 28 and 48 under hyaluronidase condition.


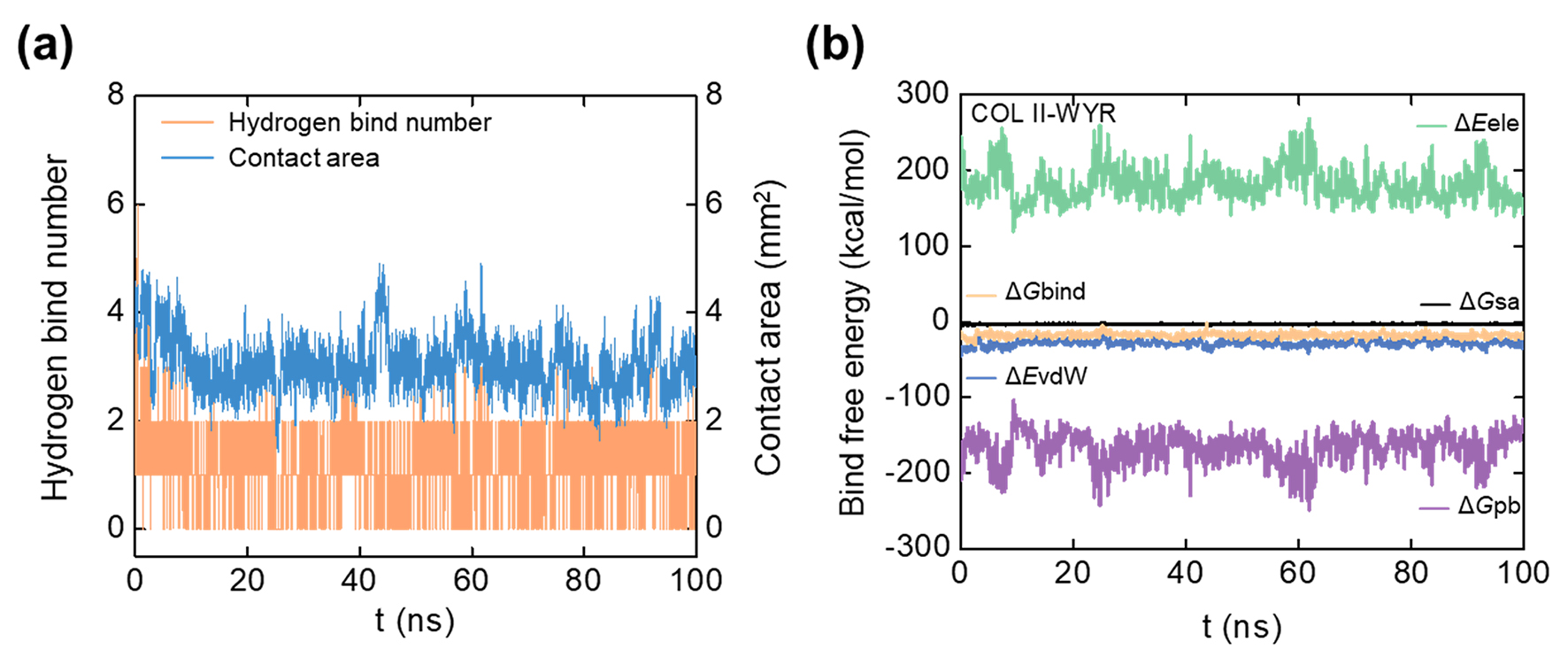


**Figure S5.** (a) The hydrogen bind number and contact area between COL II and WYR. (b) The average free energies of binding between COL II and WYR.
